# Toll-like receptors in prostate infection and cancer between bench and bedside

**DOI:** 10.1111/jcmm.12055

**Published:** 2013-04-04

**Authors:** Guido Gambara, Paola Cesaris, Cosimo Nunzio, Elio Ziparo, Andrea Tubaro, Antonio Filippini, Anna Riccioli

**Affiliations:** a Istituto Pasteur-Fondazione Cenci Bolognetti, Department of Anatomy, Histology, Forensic Medicine and Orthopaedics, Section of Histology and Medical Embryology, Sapienza University of RomeRome, Italy; b Department of Biotechnological and Applied Clinical Sciences, University of L'AquilaL'Aquila, Italy; c Department of Urology, Sant'Andrea HospitalRome, Italy; d Department of Urology, Sant'Andrea Hospital – Department of Clinical and Experimental Medicine, Sapienza University of RomeRome, Italy

**Keywords:** prostate carcinoma, prostate infections, TLR, clinical trials, cancer therapy, innate immunity

## Abstract

Toll-Like receptors (TLRs) are a family of evolutionary conserved transmembrane proteins that recognize highly conserved molecules in pathogens. TLR-expressing cells represent the first line of defence sensing pathogen invasion, triggering innate immune responses and subsequently priming antigen-specific adaptive immunity. *In vitro* and *in vivo* studies on experimental cancer models have shown both anti- and pro-tumoural activity of different TLRs in prostate cancer, indicating these receptors as potential targets for cancer therapy. In this review, we highlight the intriguing duplicity of TLR stimulation by pathogens: their protective role in cases of acute infections, and conversely their negative role in favouring hyperplasia and/or cancer onset, in cases of chronic infections. This review focuses on the role of TLRs in the pathophysiology of prostate infection and cancer by exploring the biological bases of the strict relation between TLRs and prostate cancer. In particular, we highlight the debated question of how reliable mutations or deregulated expression of TLRs are as novel diagnostic or prognostic tools for prostate cancer. So far, the anticancer activity of numerous TLR ligands has been evaluated in clinical trials only in organs other than the prostate. Here we review recent clinical trials based on the most promising TLR agonists in oncology, envisaging a potential application also in prostate cancer therapy.

- Introduction
- Toll-like receptor family as a key activator of immune response against pathogens and tumour cells
- Role of TLRs in the defence against prostate infections
- Overview of TLRs as biomarkers for prostate cancer risk
- Dual role of TLRs in prostate cancer cells
- TLR agonists as new anticancer therapy in experimental models and in clinical trials
- TLR3 stimulation in prostate cancer as a new promising therapy
- Concluding remarks and clinical perspective

## Introduction

Prostate cancer (PCa) is the second most frequent diagnosed cancer among men worldwide, accounting for 14% of total new cancer cases, and it is the sixth leading cause of cancer-related deaths, 6% of the total cancer deaths in 2008 1. Standard therapy, consisting in surgical excision of the prostate followed by androgen deprivation, initially leads to regression of the disease. Which however is often transient and no cure is known for metastatic and androgen-refractory prostate cancer. Consequently, many efforts are being made to identify novel targets for the prevention and treatment of this disease. Approximately 15% of all human cancers in adults can be attributed to infections resulting in chronic inflammation 2. The relationship between inflammatory disorders of the prostate, benign prostatic hyperplasia (BPH) and malignant tumours (PCa) is so far elusive. Epidemiological data suggesting odd ratios (OR) of 1.6–1.8 have been described for the risk of PCa in patients with prostatitis 3, while data on prostate biopsies suggest the opposite (OR 0.20 for patients with inflammatory infiltrates on prostate biopsy to have PCa) 4. Data on prostate biopsies might on the other hand be misleading with regard to the relation between inflammation and cancer because indications for biopsy mainly consist of elevated PSA, but high levels of PSA may be associated with both PCa and inflammatory disorders or infections of the prostate and the two conditions are often mutually exclusive. In BPH patients, inflammatory aspects are observed in 30–43% of cases on histological examination 5. A possible causative role of inflammation for the development of PCa is suggested by the identification of several genes which are involved in both PCa and inflammatory-related pathways (RNASEL, MSR1, GST-P1, GDF15, TLR4, TLR1-6-10. MIC1, IL1RN, IL8, IL10) 6.

Moreover, the oxidative stress associated with infection and inflammation has also been regarded as a possible cause of prostate carcinogenesis because the induction of iNOS (inducible nitric oxide synthase) might activate reactive nitrogens and oxygen reactive species that are released during the inflammatory response. Consequently, COX-2 inhibitors have been investigated for their ability to reduce the risk of developing PCa as well as other neoplasms 7.

Research on TLRs is shedding some new light on the relation between infection and PCa as the activation of the TLR family can induce an adaptive immune response against cancer and there is no better example in urology than the therapeutic effect of Bacillus Calmette Guerin (BCG) on urothelial neoplasms (provided activation of TLR 2/4 and 9 is confirmed as a crucial event in the prevention of bladder cancer recurrence) 8, 9. This is a paradigm change in the way we look at the relation between benign and malignant disorders of the prostate and is certainly worthwhile exploring.

## Toll-like receptor family as a key activator of immune response against pathogens and tumour cells

Toll-like receptors are a family of transmembrane proteins that recognize pathogen associated molecular patterns (PAMPs), molecules highly conserved in bacteria, viruses, fungi and parasites essential for their survival. TLRs are expressed on the membranes of epithelial cells, lymphocytes and antigen presenting cells, mainly dendritic cells (DCs) and macrophages, and recognize microorganism molecules (proteins of bacterial wall, nucleic acids, etc.) thus activating the onset of inflammation 10.

To date, 10 functional TLRs have been identified in humans and their ligands are lipoproteins derived from Gram-positive bacteria (recognized by TLR1, TLR2 and TLR6), lipopolysaccharide (LPS) derived from Gram-negative bacteria (TLR4), flagellin (TLR5), double-stranded RNA (dsRNA; TLR3) and single-stranded RNA (ssRNA; TLR7 and TLR8) derived from viral genome, unmethylated CpG DNA derived from bacterial or viral genome (TLR9) 11, while human TLR10 is so far an orphan receptor without a known agonist or function 12. TLRs are localized on the cellular plasma membrane, except for TLR3, 7, 8 and 9 that are localized on intracellular organelle membranes 13. TLR signalling pathways can be largely classified as either MyD88-dependent pathways, which result in the induction of inflammatory cytokines through MAPKs and NFκB activation, or TRIF-dependent pathways, which are responsible for the induction of inflammatory cytokines as well as type I interferons by IRF-3 activation 14.

Toll-Like receptors are the key sensors of the innate immunity and are critically involved in priming the adaptive immune response necessary for killing invading pathogens 15. Pathogen-led TLR activation provides rapid recruitment of inflammatory cells to the site of infection and activates them to induce an arsenal of antimicrobial functions, collectively called ‘innate immunity’ 16. TLR signalling simultaneously induces the maturation of dendritic cells, which is responsible for alerting induction of the second line of host defence, so-called ‘adaptive immunity’ 17. Thus, the innate response to a pathogen, mediated by cytokine and chemokine secretion, can be decisive in determining the nature and magnitude of the adaptive immunity 15.

Considering the essential role of TLRs in leading the innate immune response and in priming the adaptive immunity, a tight negative regulation of their signalling is crucial to avoid over-activation of the immune system resulting in acute and chronic inflammatory disorders and autoimmune disease 18. The first level of regulation is based on the decoy effect of soluble TLR isoforms (sTLR) 19, the second one on the presence of intracellular negative regulators that can block the TLR signal transduction 20. In addition, the control of TLR signalling at the level of the expression of the receptors or components of TLR signalling pathway represents another obvious strategy to regulate the immune response. Finally, the activation of TLRs can also induce apoptosis in macrophages and in epithelial cells through different signalling pathways, indicating that the immune system can be drastically shut off as *extrema ratio* 21.

Also the crosstalk with other pathways, such as the cAMP-dependent pathway, can participate to the regulation of TLR-induced signalling. It has been demonstrated that in macrophages TLR4-mediated TNF-α production is suppressed by cAMP-dependent protein kinase (PKA) 22. Moreover, it has been reported that in neonatal monocytes adenosine activates A3 adenosine receptor consequently inhibiting TLR-mediated TNF-α synthesis *via* cAMP 23.

MicroRNAs provide a tight regulation of TLR signalling at different levels: firstly regulating TLR expression itself 24, secondly controlling the expression of signalling molecules involved in TLR signal transduction and finally through the targeting of cytokine mRNAs 25. In this scenario, miRNAs could also promote deregulation of cytokine expression affecting the ability of innate immunity to prime the adaptive immune system 26. Focusing on miRNA and TLR signalling in cancer, it was recently demonstrated that TLR9 stimulation increases the growth and metastatic features of lung cancer cells *via* the downregulation of miR-7 and the resulting regulation of phosphoinositide-3-kinase regulatory subunit 3 (PIK3R3)/Akt pathway 27. Moreover, Fabbri and co-workers identified a new mechanism that links miRNAs to TLR signalling. In detail, miR21 and miR29a, secreted within exosomes by the tumour, were able to bind directly TLR7 in immune cells thus priming a pro-metastatic inflammatory response in a murine metastatic model of lung cancer 28.

In addition, TLRs can also be stimulated by endogenous molecules, such as high-mobility group box 1 (HMGB1), heat shock proteins (HSP60 and HSP70), uric acid and components of the extracellular matrix 29 in accordance with the ‘danger hypothesis’ proposing that TLRs are able to sense danger signals (danger associated molecular patterns, DAMPs) even if they originate from self proteins released from cells undergoing unprogrammed necrotic death 30 or from tumour cells treated with anticancer agents 31. Striking evidence from mouse experimental models indicates that some anticancer agents could favour the activation of immune effector cells by inducing ‘tumor immunogenic cell death’ 32. Tumour cells undergoing immunogenic cell death are characterized by the early surface exposure of calreticulin 33 and HSPs and by the late release of HMGB1. Consequently, HMGB1 acts through TLR4 expressed in DCs increasing their capability to present antigens of dying tumour cells. In a recent study, anticancer drugs capable of inducing immunogenic cell death even in human tumour cells were identified 34. The relevance of TLR4 in immunogenic cell death is further illustrated by the finding that breast cancer patients with the TLR4 allele variant, which reduces the affinity of TLR4 for HMGB1, have a higher incidence of metastasis after conventional treatments than patients with the wild-type allele 35. Moreover, these results have been confirmed also in three established tumour mouse models, in which TLR4 was consistently required to prevent tumour outgrowth upon systemic chemotherapy or local radiotherapy 31.

## Role of TLRs in the defence against prostate infections

The most evolutionarily conserved role of TLRs in host defence is the regulation of antimicrobial responses by epithelial cells, the first line of defence at mucosal sites such as the respiratory, gastrointestinal and genitourinary tracts and the skin. Nevertheless, the widely accepted hypothesis is that non-sterile sites (*i.e*. mouth, colon, or vagina) would require a response system different from that of sterile sites (bladder, kidney, prostate and testis) 36. It is conceivable that the pattern of expression of TLRs would then differ at sterile *versus* non-sterile sites and that at non-sterile sites epithelial cells might be less efficiently reactive than at sterile sites where even a low load of deleterious microorganisms should be rapidly detected and eliminated. Accordingly, many pathogens have been demonstrated to induce a robust inflammatory response in the prostate. This group of pathogens includes both ascending bacteria from infected urine, mostly *Escherichia Coli*, and sexually transmitted micro-organisms. These are bacteria and protozoa such as *Neisseria gonorrhoeae*, *Chlamydia trachomatis*, *Trichomonas vaginalis* and viruses such as *papillomavirus*, *cytomegalovirus*, *herpes simplex virus type II* and *herpesvirus 8* 37. The functional attitude to sense pathogens in male accessory glands is crucial to prevent or attack ascending infections. The pathophysiology underlying *Chlamydia* infection has been extensively studied using an experimental model of genital tract infection in mice with *Chlamydia muridarum*, a murine pathogen closely related to *Chlamydia trachomatis*. It has been shown that rat primary prostate epithelial cells (PPEC) are susceptible to *Chlamydia muridarum* infection and that they respond by up-regulating nitric oxide and chemokine production through TLR2 and TLR4 recruitment 38. In addition, in the same prostate cells it was demonstrated that even administration of the TLR4 ligand LPS alone can induce the above described proinflammatory response 39. Subsequently, Mackern-Oberti *et al*. observed that prostate epithelial/stromal cells and prostate resident leucocytes responded to Chlamydia infection through TLR signalling, which is necessary for the production of different chemokines 40.

The role of infections in BPH and PCa might be underestimated because of a number of reasons. Bacterial prostatitis is estimated to account for only 5–10% of prostatitis cases 41, but clinically the most common ‘non bacterial’ prostatitis mimics chronic bacterial prostatitis and some evidence indicates the involvement of micro-organisms that are difficult to culture 42. Moreover, chronic weak inflammation caused by a chronic infection might result in asymptomatic prostatitis escaping diagnosis. Despite these indications linking chronic prostate infections and inflammation to the development and progression of BPH and of PCa, a causative relation remains to be ascertained 6.

## Overview of TLRs as biomarkers for prostate cancer risk

Although TLRs play a central role in the host cell recognition and in the response to pathogens, recent advancement in cancer immunobiology highlights these receptors as crucial actors involved in tumour growth and progression. TLR expression is deregulated in cancerous epithelial tissues compared with tissue derived from healthy individuals, suggesting that mutations or alterations in TLR genes could be suitable markers for cancer risk evaluation, early diagnosis, or cancer patients stratification 43.

Focusing on PCa, the first evidence of a possible involvement of TLRs in cancer came from epidemiological studies 44, 45. As for TLR expression, a significant difference in TLR 4, 5, 7 and 9 in PCa tissues compared to BPH was observed by RT-PCR analysis 44. Recently, an immunohistochemistry and qRT-PCR-based screening on 133 selected patients with prostate adenocarcinoma showed association of high expression of TLR3, 4 and 9 with PCa recurrence 46. Conversely, a previous study reported that TLR3 was down-regulated in a subset of PCa samples compared with benign tissues and such downregulation was associated with higher recurrence 47. Moreover, another immunohistochemistry study performed on 62 prostate adenocarcinoma and 45 BPH samples showed that TLR9 expression was significantly increased in epithelium and stroma of PCa compared to BPH 48.

On the other hand, several studies have analysed the association of TLR single nucleotide polymorphisms (SNPs) and the risk of developing PCa 49, but the results are controversial. Recently, a comprehensive overview of these studies was published by Kutikhin and co-workers, concluding that polymorphisms in TLRs and TLR-pathway genes do not play a major role in PCa aetiology, although some of them may contribute to cancer risk assessment in specific populations 50. In this regard, Mandal *et al*. recently found that the polymorphism in TLR2 gene seems to be associated with increased risk of PCa in North Indian population 51.

The controversial results obtained in the described TLR expression profile studies as well as TLR SNP analysis could result from a variety of factors. Firstly, the possibility that micro-organisms colonized the analysed organs through latent infections was not taken into consideration. This condition would possibly lead to up-regulation of TLR expression linked to the host defence response, but not necessarily to cancer outbreak. Secondly, in some of these expression profile reports, the number of patients recruited was considerably low, possibly impairing validation across independent data set. A third possible reason for the discrepancies in the reported observations include differences in the sensitivity, specificity and/or reproducibility of assays and epidemiological sources of bias, such as confounding selection, and reverse causality biases. Certainly further investigation is needed to definitely determine the role of specific TLR polymorphisms and TLR expression deregulation in prostate pathologies.

## Dual role of TLRs in prostate cancer cells

Toll-Like receptor ligands, PAMPs or synthetic compounds could have great potential as novel anticancer agents 43. On the other hand, inhibiting certain specific TLRs in inflammation-associated cancers might yield new therapies 52.

The first investigation on the effects of TLR stimulation in PCa was performed in the epithelial cell line PC3, derived from bone metastasis of human prostate adenocarcinoma. It has been shown that PC3 cells express TLR2, and that membranes of *Mycoplasma hominis* activate NF-kB leading to secretion of the inflammatory cytokine IL-8 53. Moreover, it has been reported that rat prostate adenocarcinoma derived MAT-LU cells constitutively express TLR4 and respond to the TLR4 ligand LPS through the activation of ERK1/2 and NF-kB, up-regulating numerous chemokines such as MCP1, MIP1a, IP10, RANTES and IL-8 39. Subsequently, Andreani and co-workers showed that LPS stimulation of MAT-LU cells *in vitro*, before inoculation, inhibited tumour growth in syngeneic rats but not in athymic nude mice, indicating that TLR4 stimulation can elicit the T lymphocyte-mediated immune response against the tumour rather than directly acting on PCa cells 54. Conversely, TLR4 knock-down in PC3 cells resulted in a dramatic reduction of tumour cell viability and invasion 55. Intriguingly, in accordance with a pro-tumoural role of TLR4 in PCa, it was recently reported that the TLR4 ligand peroxiredoxin-1 is over-expressed in human PCa specimens and that it regulates prostate tumour growth in a murine cancer experimental model through TLR4-dependent induction of prostate tumour vasculature 56.

TLR9 is highly expressed in LNCaP and C4-2B cells while in PC3 and Du-145 this receptor is moderately expressed. The TLR9 ligand CpG-motif containing unmethylated oligonucleotides (CpG-ODN) and bacterial DNA induced an increased invasion of PCa cells *via* MMP-13. Surprisingly, CpG-ODN decreased the viability of all the TLR9^+^ prostate cell lines analysed. Moreover, considering the subcellular localization of TLR9 in acidic organelles, chloroquine, an inhibitor of endosome-lysosome acidification, was tested and proved capable of abolishing the invasion of PCa cells 57. It has been shown that, in primary and immortalized prostate epithelial cells expressing TLR9, CpG leads to a dose dependent increase in the proliferation rate, activation of NF-kB and increased resistance to TNF-alpha-induced apoptosis 58. These data highlight the double-edge sword feature of different TLRs stimulation and suggest that molecules involved in TLR signalling pathways might represent new targets to directly inhibit tumour growth or to improve immunotherapy in PCa after thorough screening.

## TLR agonists as a new anticancer therapy in experimental models and in clinical trials

The use of specific TLR agonists alone or in combination with standard chemo- or radio-therapy has been shown to represent a valid anti-cancer strategy in different *in vitro* or *in vivo* cancer models and several molecules have been tested in clinical trials (http://www.clinicaltrials.gov) 59, 60.

The first evidence of anti-cancer activity of pathogen-derived molecules came from William Coley's studies showing that gram positive/negative-inactivated toxins had a relevant effect in cancer treatment 61. It was subsequently demonstrated that Coley's toxin components (bacterial proteins, lipids and DNA) induced stimulation of TLRs. Since then, a large number of pathogen-derived drugs or synthetic compounds capable of selectively stimulating TLRs have been developed 60. To understand the effect of these molecules on the inhibition of tumour growth, the activity of TLR agonists has been evaluated alone, in combined therapy with other cytotoxic drugs or as vaccine adjuvants.

The poly-TLR agonist, Cadi-05, has been shown to reduce the growth of murine myeloma and thymoma in mice 62. This compound has also been used in clinical trials for the treatment of prostate and bladder cancers (NCT00525408 and NCT00694915: the recruitment status of this study is not known because the information has not been verified recently), and melanoma (NCT00675727) which was voluntarily terminated because its efficacy as a single agent in this patient population was unlikely.

Lipid-A, the active component of LPS, and other lipid-A –derived synthetic molecules, such as OM-174, are TLR4 agonists capable of reducing tumour growth in the murine B16 melanoma experimental model through the activation of natural killers (NK) and cytotoxic T lymphocyte (CTL) mediated anti-tumoural response 63.

TLR2 activation is induced by Pam_3_CSK_4_, LTA, MALP2, SMP-105 and the last has been approved for the treatment of bladder cancer 60. Similarly, another US-FDA approved drug for bladder cancer treatment is an attenuated *Mycobacterium bovis* preparation of bacillus Calmette-Guerin (BCG) 9. Cell wall components of BCG activate TLR2/4 8 and its DNA triggers TLR9 signal transduction.

TLR3 agonists used in clinical trials have shown controversial efficacy. The TLR3 agonist poly A:U proved effective in the treatment of operable breast cancer 64 while it was ineffective in a double blind trial for resectable colorectal cancer 65. Recently, Salaun *et al*. demonstrated that patients with breast cancer overexpressing TLR3 are sensitive to dsRNA anticancer therapy and that the synthetic analogue of dsRNA poly A:U injected in immunodeficient mice inhibits the growth of breast cancer and melanoma xenografts 66.

It has been shown that the TLR5 agonist flagellin, derived from Salmonella, combined with CpG oligonucleotides, induces inhibition of tumour growth in a mammary cancer mouse model 67. Moreover a TLR5 synthetic agonist, CBLB502, showed radio-protective effects only in non-transformed cells of mouse and in primate experimental models, opening a new perspective in the use of TLR5 agonists as adjuvants of radiotherapy 68. A phase I clinical trial to assess the safety and tolerability of the TLR5 agonist CBLB502 is recruiting patients with locally advanced or metastatic solid tumours. The second objective of the study is to assess the preliminary evidence of the efficacy of this molecule and to correlate the naive tissue expression of TLR5 with the clinical response and the levels of cytokines induced by CBLB502 treatment (NCT01527136).

TLR7 and TLR8 known to bind ssRNA deriving from viral genome and synthetic sequences with single nucleotide substitution allow to selectively activate TLR7 and/or TLR8 response. It has been shown that a synthetic imidazoquinoline, Imiquimod, specifically targets TLR7 inducing innate and adaptive immunity response and cancer cell apoptosis in primary skin tumours and cutaneous metastases 69. Imiquimod was effective in a Phase III clinical trial in patients with superficial basal cell carcinoma 70 and, in another clinical trial, in a subset of patients with breast cancer metastatic to skin/chest wall 71. In a phase II study in patients with advanced melanoma the systemic administration of another TLR7 agonist, 852A, induced immune activation and disease stabilization in a subset of patients 72.

Unmethylated CpG islands found in bacterial DNA are known to bind TLR9. Synthetic CpG oligonucleotides are also called Immunomodulatory Oligonucleotides (IMOs); IMOs have been successfully applied alone or in combination with chemotherapy in different cancer mouse models 73. The synthetic TLR9 agonist PF-3512676 has been successfully used in combination with taxane/platinum therapy in a phase II trial for advanced non-small cell lung Cancer (NSCLC), but a subsequent phase III study showed that this molecule failed to enhance the anticancer effect of chemotherapy and increased its toxicity 74, 75. The PF-3512676 anticancer activity has been also described in two phase II clinical trials for low grade B-cell lymphoma and metastatic melanoma 76, 77, but further phase III trials are needed to confirm the value of this therapeutic approach. IMO-2055 is currently used in combination with bevacizumab and erlotinib in clinical trials recruiting patients with NSCLC (NCT00633529), and in combination with cetuximab and irinotecan in patients with colorectal cancer (CRC) (NCT00719199) (clinicaltrials.gov). Table 1 summarizes clinical trials that include a TLR-based therapy.

**Table 1:** Clinical trials including a TLR-based therapy in patients with different tumours

Molecule	TLR target	Cancer	Phase	Reference no.
VTX-2337	TLR-8	Squamous cell cancer of head and neck	Phase I	NCT01334177
Resiquimod (R848)	TLR-7 TLR-8	Melanoma	Phase II	NCT00960752
Imiquimod (IMQ)	TLR7	Breast cancer	Phase I/II	NCT01421017
CBLB502	TLR5	Solid tumor	Phase I	NCT01527136
VTX-2337	TLR8	Fallopian tube cancer Ovarian cancer Primary peritoneal cavity cancer	Phase I	NCT01294293
VTX-2337	TLR8	Low grade B cell lymphoma	Phase I/II	NCT01396018
852A	TLR7	Breast cancer Ovarian cancer Endometrial cancer Cervical cancer	Phase II	NCT00319748
Ampligen	TLR3	Ovarian cancer Fallopian tube cancer Primary peritoneal cancer	Phase I/II	NCT01312389
Poly-ICLC	TLR3	Melanoma	Phase I/II	NCT01079741
CpG 7909	TLR9	Lymphoma, non-Hodgkin	Phase I/II	NCT00185965
CpG 7909	TLR9	Esophageal cancer	Phase I/II	NCT00669292
PF-3512676	TLR9	Non-small cell lung cancer	Phase II	NCT00321815
IMO-2055	TLR9	Non-small cell lung cancer	Phase I	NCT00633529
IMO-2055	TLR9	Colorectal cancer	Phase I	NCT00719199
EMD 120108	TLR9	Squamous cell carcinoma of the head and neck	Phase II	NCT01040832
VTX-2337	TLR8	Low grade B cell lymphoma	Phase I/II	NCT01289210
EMD 1201081	TLR9	Squamous cell carcinoma	Phase II	NCT01040832
BCG	TLR2/4	Bladder cancer	n.s.	38
polyA:U	TLR3	Breast cancer	n.s.	39
Imiquimod	TLR7	Superficial basal cell carcinoma	Phase III	43
Imiquimod	TLR7	Breast cancer skin metastasis		44
852A	TLR7	Melanoma	Phase II	45
PF-3512676	TLR9	NSCLC	Phase III	47

TLR: Toll-Like receptors; NSCLC: non-small cell lung Cancer; BCG: Bacillus Calmette Guerin.

At present, there are no trials using TLR agonists for PCa although it may represent an ideal candidate and very neat model. PCa goes through an initiation phase that probably occurs relatively early in the patient adult life and a progression phase that may lag for years 78. The immune adjuvant activity of TLRs makes them ideal candidates to treat low tumour burdens when a few transformed cells should be eliminated or their number should be controlled to maintain the tumour below the threshold of clinical relevance. Various scenarios involving PCa can be identified as suitable target for TLR-based therapy: patients with diffused high-grade prostatic intraepithelial neoplasia (HGPIN), patients with atypical small acinar proliferation (ASAP), patients with low risk PCa which are currently enrolled in active surveillance programmes, patients receiving radiation therapy for localized prostate tumour, patients at risk of local tumour recurrence.

## TLR3 stimulation in prostate cancer as a new promising therapy

Interestingly, although conflicting reports have been published concerning the pro- or anti-tumoural role of several TLRs, literature data agree on an anti-tumour role for TLR3 in various cancers. In fact, the TLR3 ligand Ampligen has been proposed as a potentially safe immune-adjuvant in cancer therapy 79.

We have demonstrated that the stimulation of TLR3 by means of the synthetic ligand Poly (I:C) leads to different effects on two human PCa cell lines, LNCaP and PC3. Poly (I:C) inhibited the proliferation and induced apoptosis in both cell lines, with much higher efficiency in the former than in the latter more aggressive line, depending on differential degree of upregulation of the powerful tumour shield, hypoxia inducible factor-1 (HIF-1) 80, 81. In the light of these results, we proposed a combination of HIF-1 inhibitor and TLR3 agonist for the treatment of solid tumours (patent pending PCT/EP2011/056006). Moreover, the anti-cancer effect of HIF-1α inhibitors is not limited to its direct activity on prostate cancer cells: in fact considerable evidence suggests that other stromal components, *in primis* immune cells, are influenced by hypoxic environment with consequences on tumour growth 82. It has been shown that in T lymphocyte HIF-1α plays an anti-inflammatory and tissue-protective role by negatively regulating T cell function 83. Interestingly, the knock-out of the 1.1 isoform of HIF-1α (<30% of the total) significantly increased T cell activation by enhancing TCR-induced cytokine production 84, suggesting a crucial role of this specific isoform in this process. Such immunosuppressive activity of HIF-1α could play a detrimental role in tumour microenvironment inhibiting the anti-tumoural immune response of T cells.

**Fig. 1:**
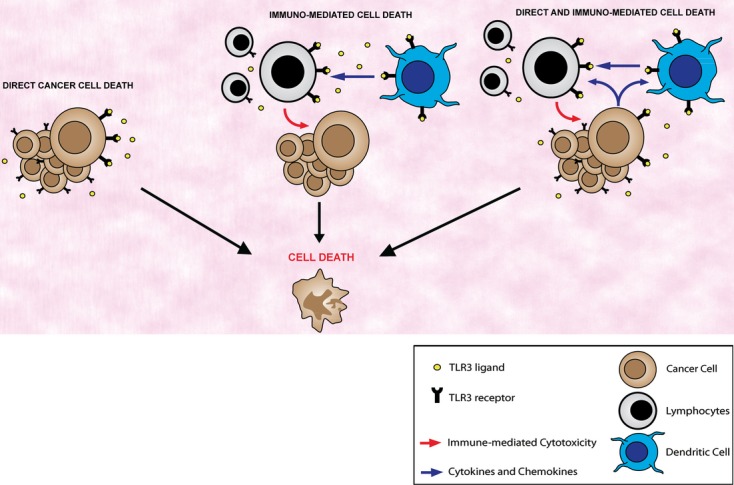
Different strategies for anti-tumour TLR3 activity. The direct effect of TLR3 ligands on PCa cells consists of tumour growth inhibition and cancer cell apoptosis (direct cancer cell death). Alternatively, engagement of TLR3 receptors, mainly expressed on the immune system cells, could result in immune-mediated tumour growth suppression (immune-mediated cell death). TLR3 ligands could stimulate their receptors both in cancer cells and in immune cells inhibiting tumour growth both directly and through the immune system (direct and immune-mediated cell death). Original cartoon.

The direct apoptotic effect of TLR3 stimulation was only partially confirmed in a subsequent study in which the authors evaluated the effect of poly (I:C) on TRAMP-C2 murine PCa cells transplanted in syngeneic mice, showing that the suppression of tumour growth induced by poly(I: C) was dependent on T-lymphocyte and NK cells recruitment in the tumour microenvironment. The authors described an interferon-I-dependent mechanism in which NK cells inhibited the immune-suppressive T regulatory lymphocytes, favouring the anti-tumour immune response 85. Collectively, these data suggest three different hypothetic functioning modes for TLR3-dependent anti-cancer mechanism in PCa (Fig. 1). Briefly, in the first hypothesis the direct effect of TLR3 ligand on PCa cells would induce tumour growth inhibition and cancer cells apoptosis (direct cancer cell death). In the second, TLR3 receptor, mainly expressed on the immune system cells, could induce immune-mediated tumour growth suppression (immune-mediated cell death). Finally, TLR3 ligands could stimulate their receptors both in cancer cells and in immune cells promoting the inhibition of tumour growth both directly and through an immune-mediated mechanism (direct and immune-mediated cell death). In accordance with the third model, our group has previously demonstrated that activation of TLR3 in PCa cell lines induces the secretion of cytokines and chemokines that could recruit and activate immune cells in the tumour site consequently promoting their anti-cancer activity 86. In this view, the activation of TLR3 on the membrane of cancer cells could act as a trigger for the immune response against cancer (Fig. 1) and, by inducing both tumour cell death and anticancer immune stimulation, could synergize for optimal immunochemotherapy in PCa.

## Concluding remarks and clinical perspective

This is an exciting time in medicine because the gap between the bench and the clinic has been bridged and new hypotheses can be tested in the laboratory, verified in animal models and confirmed in clinical trials. Research on TLRs opens a new perspective on the relation between infection and cancer development that may offer new therapeutic strategies. Tumours with a long natural history that develop in organs prone to infections, such as the prostate, may be good candidates for proof of concept and for exploring possible therapeutic interventions. For the past 30 years, treatment of PCa has been based on surgery, radiotherapy and hormone manipulation, but new insight into the molecular interaction between the host and the tumour may hopefully lead to interesting developments in this field.
